# Unfolding Crease Patterns Inspired by Insect Wings and Variations of the Miura-ori with a Single Vein

**DOI:** 10.3390/biomimetics4030045

**Published:** 2019-07-05

**Authors:** Thibaut Houette, Eric Gjerde, Petra Gruber

**Affiliations:** 1 Department of Biology, The University of Akron, 235 Carroll Street, Akron, OH 44325, USA; 2 Eric Gjerde Studio, 1122 Edmund Avenue, Saint Paul, MN 55104, USA; 3 Myers School of Art and Department of Biology, The University of Akron, 150 East Exchange Street, Akron, OH 44325, USA

**Keywords:** folding, Miura-ori, insect wings, venation system, pneumatic, biomimicry, biomimetics

## Abstract

In many disciplines, professionals are interested in folding patterns for their packing and shape changing capabilities. Many insects have folded wings fitting to their body morphology that can unfold to fly, support their weight and withstand external forces. This paper focuses on the main characteristics emerging from folding patterns inspired and adapted from both insect wings and Miura-ori patterns, along with the actuation mechanism. Pneumatic actuators, similar to the venations on insect wings, are used to unfold these patterns. Depending on one vein’s placement, its inflation can unfold models with many creases. While a single vein cannot fold the model back, a snapping behavior, observed in some folding patterns, could be used to trigger the folding mechanism of a model. By presenting the characteristics of each folding pattern studied in this work, one could come forth with an application and choose the most efficient folding patterns based on the most suitable characteristics for this application. These folding patterns can then be optimized to address specific requirements by adapting their different parameters.

## 1. Introduction

Due to their packing and shape changing capabilities, folding patterns and tessellations have been used in many architectural and engineering applications, such as drones [1] shading systems [2], and also design for outer space [3]. Tessellations can go from a flat 2D shape to a 3D object [4] with the help of simple actuations, such as pulling on opposite corners. Because of their scale-free characteristics, origami patterns can be used at various scales from nano to macrometer size models. The interest is even more pronounced for flat-foldable origami tessellations as they are flat during both folded and unfolded states, reducing their surface area for packing. Self-folding structures, actuated by environmental triggers, are also of increasing interest for architectural applications [5].

In biology, folding is mostly portrayed in insect wings, leaves and flowers. Insect wings need to fit along the insect’s body, usually under its elytra, and to lift the insect during flight. These two functions correspond to the folded and unfolded states of insect wings. Fitting along the body requires compactness in comparison to the large surface area necessary to lift the insect during flight. For example, the surface area of an unfolded hind wing of earwig (Dermaptera) is ten times greater than a folded one [6]. Therefore, insect wings usually possess a high surface area difference between the folded and unfolded states, which is of particular interest for numerous applications that might require extreme packing.

While insect wings possess many veins, researchers found that the crease pattern corresponds to the resilin distribution along the insect’s wing [6]. Resilin is a rubber-like protein [7] that stores elastic energy along the creases to facilitate the folding and unfolding mechanisms [8]. Ergo, the folding pattern of an insect’s wing can be analyzed by studying the resilin distribution.

**Figure 1:**
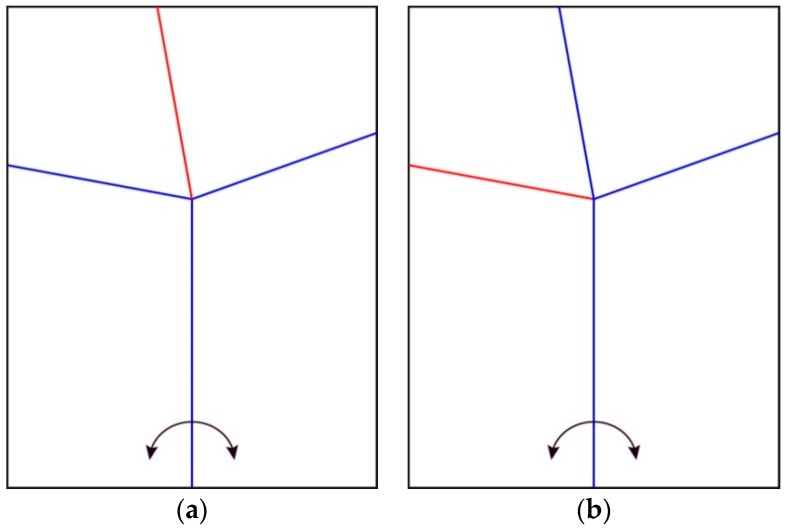
Unfolded pattern based on the drawings from Haas and Wootton’s abstraction of insect wings [9]. (**a**) and (**b**) display the two different ways of folding this pattern. The blue and red lines represent valley and mountain folds, respectively, knowing that these folds can also be inverted. The arrows at the bottom show the actuation angle to fold and unfold this pattern.

Some insect wings carry impressive transverse folding patterns to increase the packing capacity. These transverse folds are actuated remotely by other parts of the folding pattern. Therefore, these creases can be used to unfold complex patterns with a limited number of actuators. Haas and Wootton (1996) studied a variety of insect wing folding patterns, including Coleoptera, Dermaptera and Blattodea [9]. They abstracted them into a configuration that can be folded in two different ways (Figure 1). This configuration has one vertex where four creases intersect, forming four sheets or sectors. None of the creases are aligned with one another on the other side of the vertex. Because of this characteristic, this pattern can be folded and unfolded in two different ways by rotating two sheets along their common crease line.

**Figure 2:**
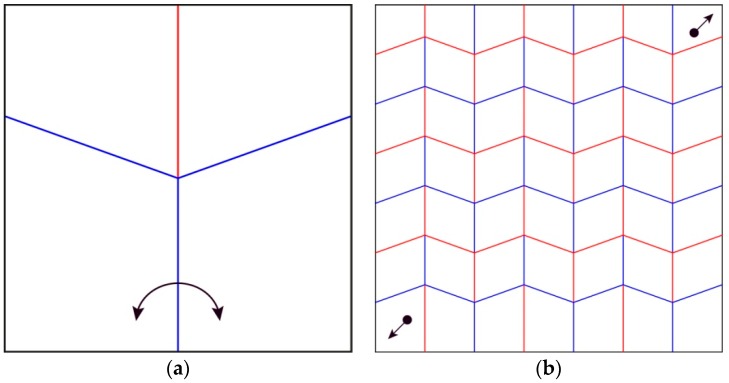
Miura-ori tessellation adapted from Miura [10]. (**a**) Shows one unit cell of the Miura-ori pattern; (**b**) presents how this cell can be duplicated. The arrows display actuation mechanisms to unfold the patterns.

The Miura-ori tessellation (Figure 2), designed by Miura for the packaging and deployment of large membranes for space missions [10] displays similar behaviors as the one abstracted by Haas and Wootton. This tessellation is well studied for many different reasons: it can be folded and unfolded flat by pushing or pulling two points of the pattern; the number of stacked edges is reduced; and it behaves as an auxetic structure, meaning that when the length of the model in one direction is reduced, the length in the perpendicular direction is also reduced. These characteristics made this folding pattern highly efficient for packing while limiting edge deterioration.

In addition to the insect wing folding patterns, the actuation mechanism is crucial for the folding function. Different mechanisms used by insects to unfold their wings have been found. Knowing that each species is slightly different, the unfolding of the wings usually results from a combination of forces. Firstly, the elasticity of the material located at the creases of the wing, called resilin, triggers the unfolding [8]. The elytron, the hard cover, blocks the wing from unfolding until the insect opens it, allowing the wing to unfold. Secondly, once the wings are partially open due to the release of the elastic tension in the material, the insect will often flap to fully open them. Less obvious, thirdly, is the unfolding mechanism through inflation of the veins due to increased liquid pressure. The first example showing this specific mechanism is when some Lepidoptera, butterflies and moths, hatch from their chrysalis. In addition to hanging upside down to allow the wings to unfurl with gravity [11], meconium, a liquid waste from the chrysalis stage, is pumped into the veins [12]. The meconium will then be excreted from the body, but a small quantity will stay and harden in the veins, increasing their rigidity. While this process only occurs at the emergence from the chrysalis in Lepidoptera, some Coleoptera increase their blood pressure in the hind wing venation system to unfold their wings before flight [13]. Sun et al. actually discovered that this blood pressure increase in the veins is proportional to the length and body mass of the insect itself. Therefore, depending on the species, different mechanisms or a combination of them are responsible for the unfolding process.

Based on this background information about insect wing folding patterns and unfolding mechanisms, this paper presents an analysis of the key characteristics of crease patterns based on drawings from the patterns of Haas and Wootton, and Miura, along with an abstraction of the venation system to unfold these patterns. These patterns were selected for their high surface area ratio between the folded and unfolded states, simplicity of pattern facilitating cell duplication, non-overlapping edges in at least one direction and availability of existing research. Different design applications focus on different characteristics, such as the ratio between the surface area of the folded and unfolded states, or the number of overlapping sheets. The future applications will potentially use a diverse range of materials. For this reason, and also to reduce complexity of the investigation, this research studied geometry as an abstract characteristic and excluded folding properties related to materials and hinge mechanisms. Therefore, based on the constraints of a specific application, one could look at the most efficient folding patterns for their specific design challenge. Another key point of this paper is to hypothesize that one vein could be enough to unfold a pattern inspired by specific insect wings and the Miura folding patterns to legitimate the use of hydraulic and/or pneumatic actuation.

## 2. Materials and Methods

Physical models were generated for experimenting with the pneumatic actuation and the folding pattern adaptations. The crease patterns presented in this section were modeled in Adobe Illustrator (Adobe Systems, San Jose, CA, USA) and produced with a laser cutter (Universal Laser Systems, Scottsdale, AZ, USA) in order to investigate physical models. An online tool was also used for simulation (The Origami Simulator by Amanda Ghassaei).

### 2.1. Investigation of Biological Role Models

**Figure 3:**
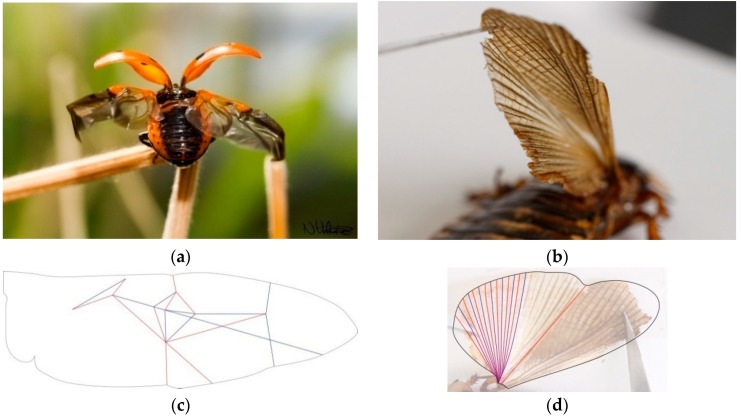
Pictures of the biological role models studied and drawings of their crease pattern on top of the unfolded state. (**a**) Presents a Coccinellidae unfolding its wings (© Flickr, Nikk, CC BY 2.0). (**b**) Image of a *Blaptica dubia* wing. (**c**) Displays the wing folding pattern of a *Coccinella septempunctata* based on drawings from Saito et al. who performed microcomputed tomographyof the wing to view the folded state. (**d**) Shows the folding pattern of the *Blaptica dubia* wing drawn over a photograph.

To understand the characteristics of folding patterns in insect wings, we studied two main organisms in addition to the existing literature: a *Coccinella septempunctata* from the Coccinellidae family of the order Coleoptera [14], commonly referred as a lady bug, and a *Blaptica dubia* from the Blaberidae family of the order Blattodea (Figure 3).

These patterns cannot be exactly folded flat for multiple reasons. To begin, the models were made with paper which has different properties than the more elastic wing materials. Furthermore, folded wings have to attach to the insect’s body, which is usually not flat. For example, the body of a Coccinellidae has a positive gaussian curvature instead of being a flat surface. Thus, the wings need to follow this specific curvature when folded. In addition, based on the microcomputed tomography performed by Saito et al., the authors have an assumption that the elytron, covering the wings, does not follow the same curvature as the insect’s body. A three-dimensional space is then created between the insect’s body and its elytra, decreasing the necessity of having a fully flat-folded wing. Therefore, when studying insect wings for human applications, the curvature of the folded state has to be taken into consideration. These patterns must be abstracted and not copied directly, as applications will not have the same functions and constraints as insect wings.

### 2.2. Duplication of the Unit Cell from the Wing-Inspired Pattern

**Figure 4:**
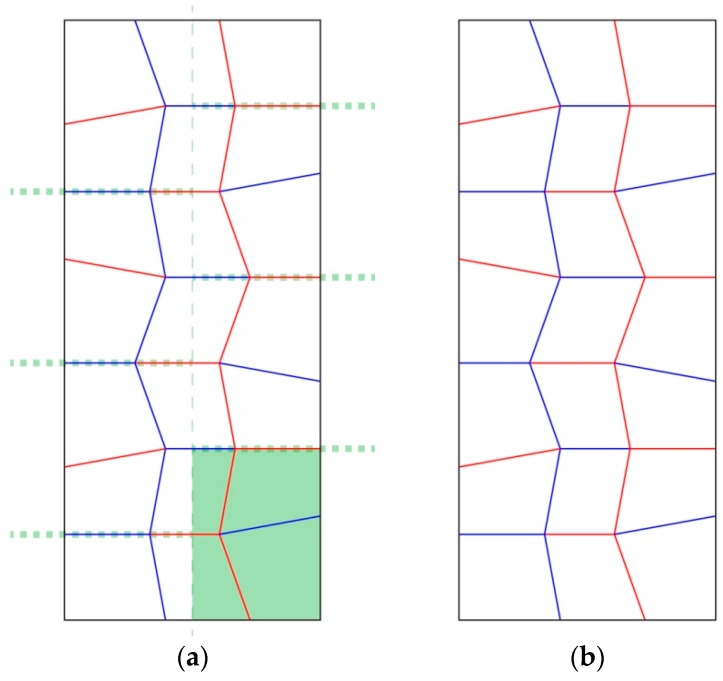
The folding pattern (#104) presented in (**b**) was made from duplicating the wing-inspired unit cell from Haas and Wootton, shown in green in the bottom right corner of (**a**). This cell was mirrored along a horizontal axis formed by the top edge of the boundary, shown as a thick green dashed line. The resulting cell was mirrored in a similar fashion to create the third cell. Then the right row of cells was mirrored along the vertical thin green dashed line and offset half a cell. While the horizontal boundary lines were kept to continue the pattern, the vertical one was deleted for this iteration. Since the transformations are applied to an entire unit cell, the angles between the creases intersecting at the node in the middle of the unit cell are identical along the pattern.

Based on existing literature regarding wing folding patterns and the discovery of trends across wing folding by experts, the authors continued the investigation with folding patterns already researched: the insect-inspired pattern by Haas and Wootton (Figure 1) and the Miura-ori by Miura (Figure 2). These two patterns originate from a unit cell, made of 4 quadrilaterals intersecting at a node. The angles between the creases intersecting at this node define how the pattern will behave when folding. The Miura-ori has an axis of symmetry, resulting in two couples of two angles equaling 180° on either side of its axis. The angles from the insect inspired pattern are all different: approximately 70°, 80°, 100° and 110°, making it asymmetrical. These folding patterns have limited properties if kept as one unit cell. To create a more complex pattern, one needs to duplicate this unit cell in order to expand their folding capacities, such as increasing the surface area distinction between the flat-folded and flat-unfolded states. Copying, rotating and mirroring transformations were applied to the original unit cell without considering the black outline since it is not actively part of the folding pattern, but just a result of fitting the unit cell in a rectangle. For some iterations, transformations were combined or applied to multiple unit cells already duplicated to produce further variations (Figure 4).

**Figure 5:**
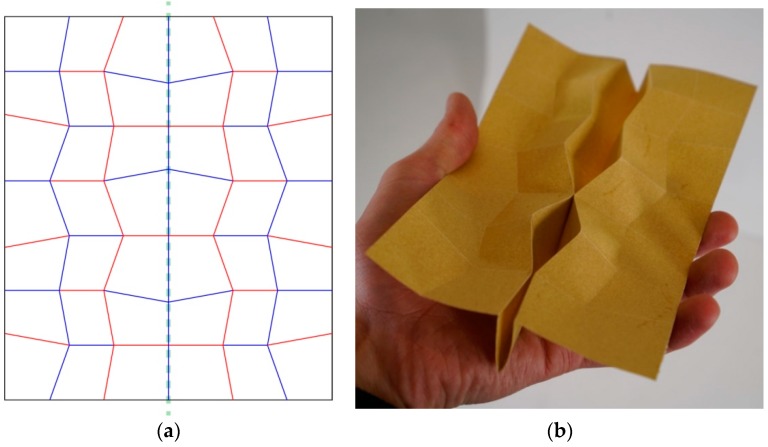
The folding pattern (#105a) presented in (**a**) was made from mirroring #104 along the green dash line and keeping this axis as a valley fold. (**b**) Displays the facing vertices or nodes blocking the folding process.

During this duplication process, some transformations block the overall pattern from being fully folded. The rules coming from the field of origami need to be respected. For example, when the pattern #104 (Figure 4) was mirrored along its left edge boundary without offsetting it, the facing vertices blocked the model from folding completely (Figure 5). While this behavior is a problem for flat foldable patterns, it could be used to maintain 3D morphologies.

**Figure 6:**
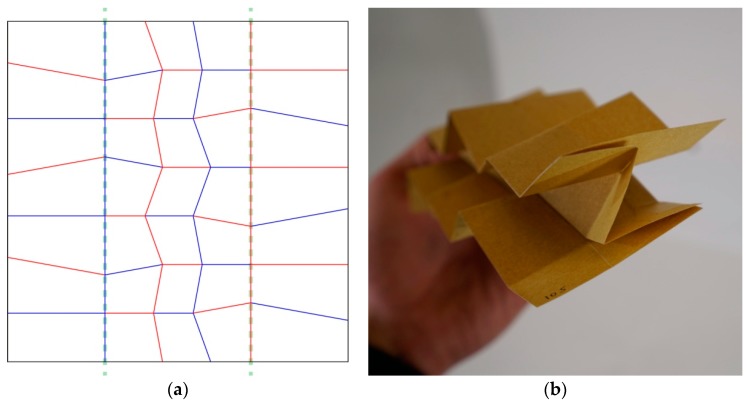
The folding pattern (#105b) presented in (**a**) was made from mirroring the last row of creases on both vertical edges of the #104 pattern, along the green dashed lines and extending them. The mountains and valleys were then inverted for these duplicated sections. This inversion unlocked the folding process, so that this pattern can be fully folded. (**b**) Presents the half-folded state of this new iteration (#105b).

This blocking problem arose because on either side of the mirrored axis, the facing creases are both mountains or both valleys. On the other hand, if the mirrored part of the pattern is flipped, meaning that the valleys would become mountains and the reverse, both sides will come on top of each other. This variation was continued in one design iteration (#105b) for one row of faces (Figure 6). These examples present the process explored during this research for the design of wing-inspired patterns.

### 2.3. Modification of the Miura-ori Pattern’s Angles

**Figure 7:**
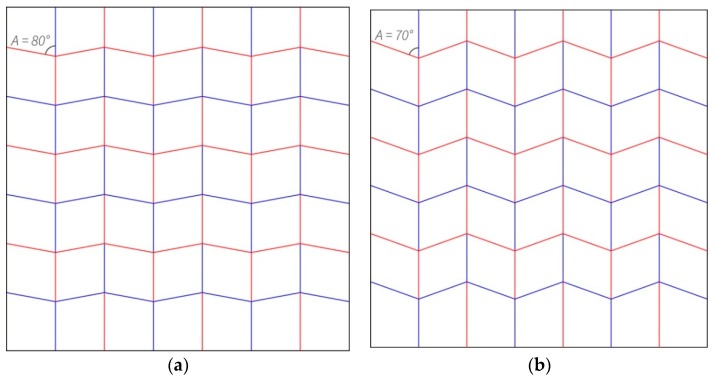
Both (**a**) and (**b**), respectively #204 and #205 for this research, are variations of the Miura-ori pattern. (**a**) Is defined by couples of 80° and 100° angles between creases intersecting at the nodes, whereas (**b**) is composed of couples of 70° and 110° angles.

**Figure 8:**
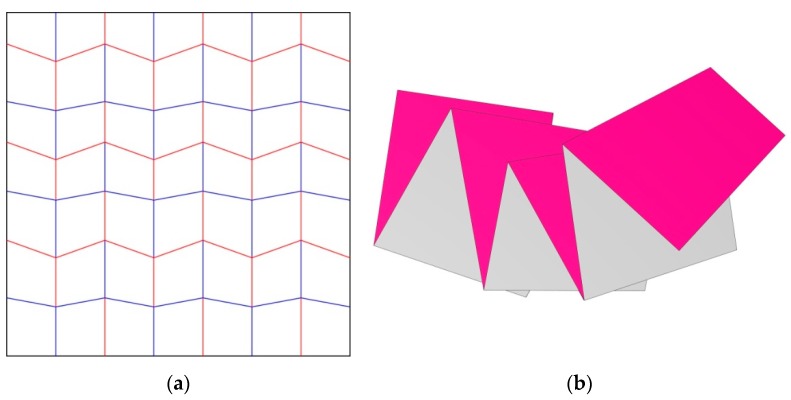
The folding pattern #206 is presented in (**a**) with its folded state in (**b**), where the curvature of the folded state can be observed. (The Origami Simulator, built by Amanda Ghassaei and accessible online, was used to produce this image, http://apps.amandaghassaei.com/OrigamiSimulator/).

The Miura-ori exploration was mainly focusing on the impact of modifying the angles between the creases intersecting at the middle node of the unit cell on the pattern’s characteristics (Figure 7). Because of the different angles between both variations, the pattern has different characteristics, such as a different ratio between the surface area of the folded and unfolded states. For #204, the folded surface area is 4.3% of the unfolded state and for #205, it is 6.0%. In order to push this research further, angle variation was introduced in one of the iterations, #206 (Figure 8). The folding of this pattern generated a curvature of the model, meaning that fewer edges were exactly stacked on top of each other.

### 2.4. Analysis of the Folding Patterns Generated

**Figure 9:**
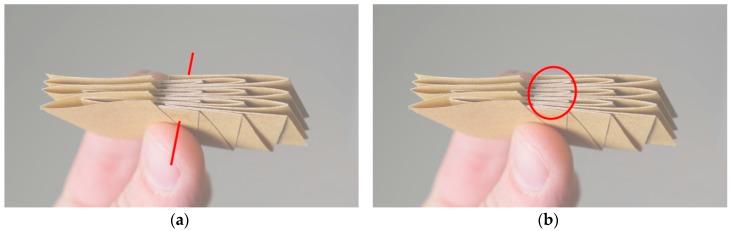
Folded state of a Miura-ori pattern. The red line in (**a**) shows the location with the highest number of overlapping sheets of paper. The red ellipse in (**b**) displays the location where the highest number of edges or creases are exactly stacked onto each other.

All of the folding patterns generated were analyzed for specific parameters in order to showcase their advantages and disadvantages. This abstraction of the patterns in terms of properties will facilitate the future selection to fit specific applications. The first parameter is the number of flat states. For example, a pattern that cannot be fully flat-folded will only have one flat state as all of the patterns explored originate from a flat surface. Secondly, the number of stable states was recorded. A stable state is defined by being at rest, where no potential energy is stored. The number of unit cells in the entire pattern was also recorded. Due to the aforementioned complications when duplicating these patterns, the number of unit cells was not the same for all of the iterations, but it is important to take this into consideration when looking at the results of this study. If the model can be folded flat, the following parameters are also analyzed: the surface area difference between the folded and unfolded states, the maximum number of overlapping sheets of material when folded flat (Figure 9a), and the maximum number of edges stacked exactly on top of each other in the folded state (Figure 9b). The surface area of the folded state was done by scanning the folded models with a scale bar. The scans were then imported and scaled into Rhinoceros 5.0 (Robert McNeel & Associates, Seattle, WA, USA), a computer-aided design software. The perimeter of this folded geometry was drawn, and its area was calculated by the software from this perimeter.

These characteristics allowed the authors to abstract the folding patterns and determine their principal properties that can be seen as benefits or drawbacks depending on the applications. With these properties displayed, one could select the pattern of interest for a specific application. After the selection, the pattern will need to be adapted to the application but will provide a reference to follow in order to achieve similar properties.

### 2.5. Implementation of a Venation System in the Models

A previous study already explored the use of inflatable tubes, inspired by insects’ wings to unfold a Miura folding pattern [15]. Senda et al. used these tubes along all of the creases in one direction, whereas this study aims at unfolding the entire pattern with the implementation of only one vein based on its placement.

The veins were produced with a 25 mm flat width dialysis tubing (Spectra/Por4, Spectrum Chemical Mfg. Corp., New Brunswick, NJ, USA). This tubing was selected because it can be fully flat and easily inflated. On the other hand, the material is not completely air- or water-tight. Because the experiments were looking at the inflation stage and not the inflation over time, this material was sufficient to observe the actuation mechanism. The inflation was produced by blowing air into the dialysis tubing. Beyond the experimental stage, it would be more efficient to use a hydraulic system instead of a pneumatic system, as water cannot be as easily compressed. In the models produced, material was removed at the crease locations where veins were present in order to facilitate the air movement throughout the tube when the model is folded.

## 3. Results

### 3.1. Folding and Unfolding Performances of the Original Patterns

**Figure 10:**
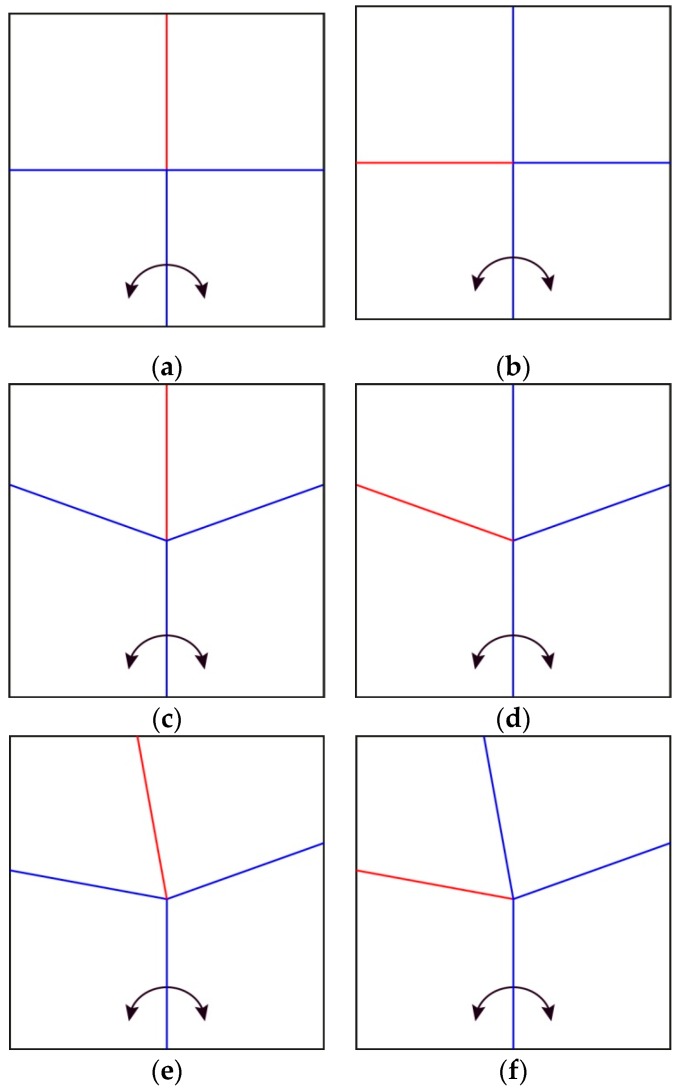
Crease patterns of the three original unit cells for this study. (**a**,**b**) Presents a folding pattern with four 90° angles meeting in one node. (**c**) Is the representation of one cell of a Miura crease pattern [10]. (**d**) Is an adaptation of a Miura-ori unit cell to a second type of folding. (**e**,**f**) are based on drawings from Haas’ and Wootton’s abstraction of insect wings. The left column (**a**,**c**,**e**) displays one type of folding where the one reversed crease is on the opposite side of the angle generating the folding and unfolding. The right column (**b**,**d**,**f**) shows the same patterns with the reversed crease on one of the sectors used to open or close the angle. The arrows present the opening angle, triggering the pattern unfolding.

As a start, three unit cells, made of four sheets were investigated for their response to the opening of an angle between two sheets. The first was made of creases perpendicular to each other intersecting at one node. The angle between each crease was 90° (Figure 10a,b), resulting in two couples of creases aligned with their opposing crease, forming two perpendicular lines. The second one (Figure 10c,d) is the Miura-ori, which has already been explained in the introduction, along with the third pattern (Figure 10e,f), based on drawings from Haas’ and Wootton’s abstraction of insect wings [9]. The unfolding and folding behaviors based on an angle opening are as follows:

- The pattern in Figure 10a can only be half unfolded and folded back.
- The patterns in Figure 10b and d can be fully unfolded but only half folded from the unfolded state.
- The patterns in Figure 10c,e and f can be fully unfolded and folded back.

The discovered trend is that when two facing creases are aligned and form a single line in the 3D space at some point of the folding or unfolding process, continuing to open or close the angle at the bottom of the pattern will not further modify the pattern besides the sectors used to open the angle. For this reason, the insect wing-inspired pattern can be fully unfolded and folded back as none of the creases are aligned at any point of the folding process.

### 3.2. Resulting Characteristics of Each Pattern Generated

**Figure A1:**
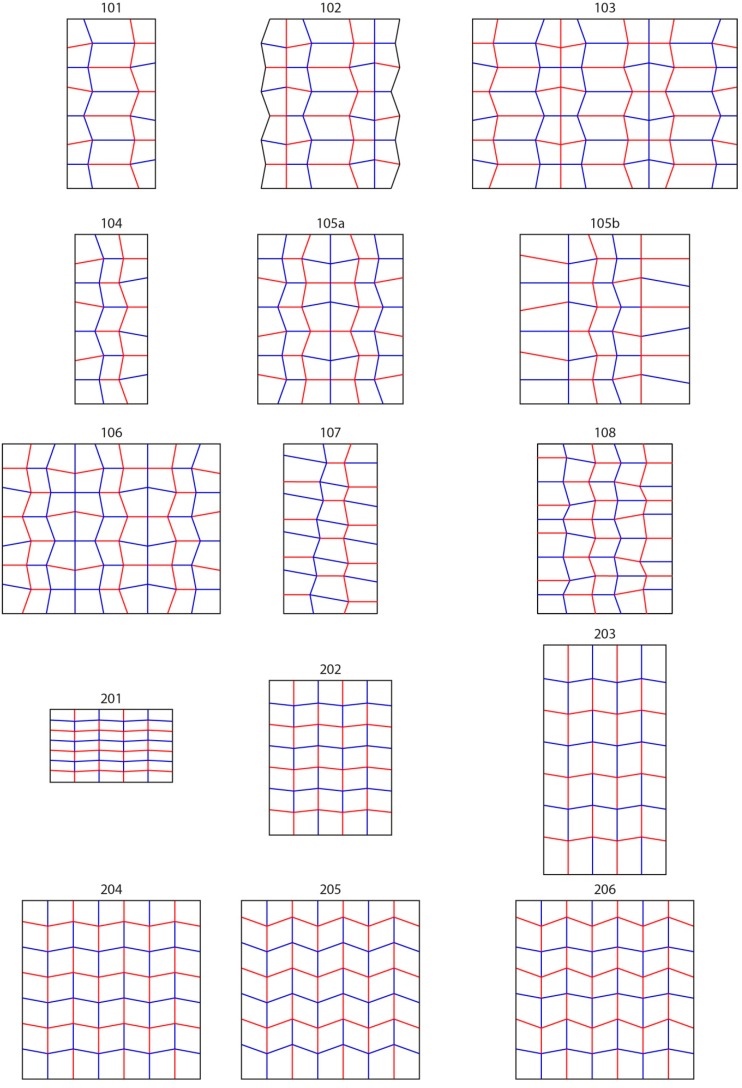
This figure shows the different patterns studied during this research. The numbers starting with a 1 are adapted from the insect wings inspired pattern. The ones starting with a 2 are variations of the Miura-ori pattern.

Table 1 presents the results of the folding patterns’ analysis generated during this research. All of the crease patterns can be seen in Appendix A (Figure A1). The number of unit cells is important to keep in mind when comparing different patterns. As a result of the transformations applied to increase the complexity of the pattern, not all of the patterns have the same number of unit cells. This pattern research is combined with the implementation of a single vein in order to unfold complex patterns. Therefore, simple patterns, made of less than 7 unit cells, were not included in this study. A potential future research direction would be to apply the same analysis to folding patterns made of increasing numbers of unit cells, until the duplication process blocks the pattern from folding completely and compare their characteristics across the various original patterns. 

While these patterns originated from only two different unit cells, each of these models has different characteristics, increasing the potential range of application depending on their constraints. For example, some applications require a pattern that can be packed down to a minimal surface area. In this case, the ratio between the surface areas of the folded and unfolded states will be a priority. On the other hand, each design has pros and cons, so the selection of an optimal pattern will require solving a trade-off between the characteristics of these patterns.

#### 3.2.1. Changing the Angle within the Miura-ori Pattern to Modify the Density of the Folded Model

For the Miura-ori variations, there were no issues with duplicating the pattern as it is a tessellation and only contains two different angles. Increasing the angle difference from 80° and 100° to 70° and 110° between the creases intersecting at the nodes resulted in a lower difference between the surface areas of the folded and unfolded states. On the other hand, the number of overlapping sheets and exactly stacked edges also decreased, due to the lower density of the folded state. The last iteration, #206, showed that when two different angle couples are introduced within a Miura-ori pattern, a curvature appears during the folding process. As a result of the curvature, the number of exactly stacked edges decreased.

#### 3.2.2. Reducing the Number of Exactly Stacked Edges with the Patterns Inspired by Insect Wings

Compared to the Miura-ori, the insect wing-inspired crease patterns produced fewer regular geometries as a result of the greater angle diversity. In this regard, the number of overlapping sheets increased, but the number of exactly stacked edges was considerably reduced. In addition, the percentages of surface areas of the folded state over the unfolded state are higher than the one studied in the Miura-ori patterns. Some of the patterns also created a snapping mechanism when a straight crease was introduced throughout the entire folding pattern as the axis of a mirror transformation of this pattern. This transformation also generated sheets exactly overlapping each other, which could increase the rigidity of the partially folded state.

### 3.3. Veins as a Pneumatic or Hydraulic Actuator to Unfold the Models

**Figure 11:**
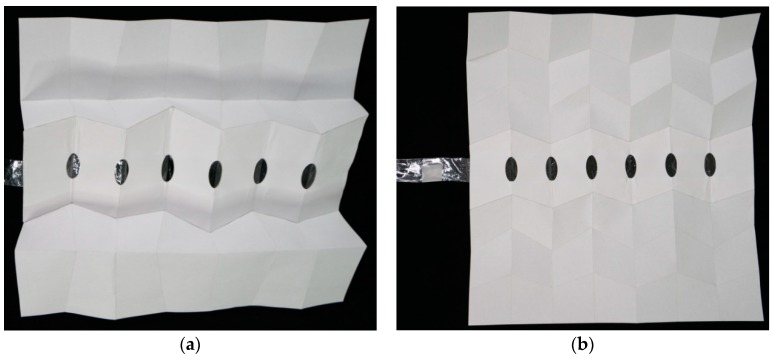
Models with the single vein implemented in the middle of the patterns. In this figure, the veins are not inflated. (**a**) Corresponds to the #105b folding pattern, while (**b**) refers to #206.

With experimental models, the authors discovered that a vein can unfold a simple pattern made of one crease either perpendicular or aligned with the vein. Since the goal is to reduce the number of veins to one, this discovery meant that one vein could cross the entire pattern, and no matter how the vein is oriented compared to the creases, it will still be able to unfold it. This characteristic increased the interest in veins as an unfolding actuator and facilitates the placement of veins to unfold the entire pattern, because some patterns need to be actuated along specific lines. Added to the experimental models, one vein was implemented in two of the models to test the efficiency of a single vein to unfold complex patterns: one adaptation of the wing-inspired patterns (#105b) and one iteration of the Miura-ori pattern (#206) (Figure 11). The vein was able to unfold the patterns in both cases, despite crossing many creases. This is possible because the patterns studied are made of unit cells actuated by opening the angle between the sheets of material (Figure 1 and Figure 2). This vein inflation will open an angle, triggering a transverse fold, which produces another angle variation, and so on. Therefore, opening a succession of angles with one vein is sufficient to unfold the entire pattern. Due to the characteristics of the paper as a material, the models did not unfold fully flat when subjected to the pressure inside the vein. Paper models were just used to show a proof of concept. For a real application, if a fully unfolded state is required, a non-flexible material should be used.

On the other hand, a vein cannot be used to fold the model from its unfolded state for two reasons. First, sucking the air out will result in the flattening of the vein without folding the model. Second, the crease closest to the air entrance may close before the other ones, resulting in some air trapped in the middle of the vein. Therefore, another system is needed to fold the model back to its original state if the pattern is not designed to stay unfolded. Some of the patterns generated produced a snapping mechanism, which folded specific parts of the models. The snapping occurred where the pattern was mirrored along an edge that was kept as a folding line. For example, in #105b presented in Figure 6, the snapping happened along the green dashed lines. If all of the creases would generate a snapping behavior, then the entire pattern could potentially close when the vein deflates.

Veins turned out to be successful actuators for the models generated. One vein, if placed in the correct location, was able to unfold the patterns. However, veins alone were not able to fold a model. While some folding patterns’ characteristics appeared logical as they relate to the pattern’s complexity and density of the folded state, unexpected characteristics also emerged from these models such as snapping, edges that are not exactly stacked on each other, and curvature in the folded state. These characteristics present opportunities for future investigation and applications.

## 4. Discussion

Numerous veins are present on insect wings because they perform multiple functions not necessarily related to the process of unfolding the wings. For example, they provide vital fluids to the different regions of the wings. This research showed that one vein can entirely unfold the patterns studied. Yet, adding more of them would strengthen the unfolded state, as it would increase the number of stiffer elements. This strength is also highly dependent on the materials used. Insect wings are extremely thin, which could be another reason behind the number of veins dispersed throughout the wing. The experimental models were made out of a combination of paper and dialysis tubing made out of cellulose filled with air as an actuator. Due to the compressive property of air, the pressure inside the veins or tubes may vary. For example, a compressed air engine would need to be used to achieve the full inflation of the vein. Using water instead would increase the efficiency and control of the unfolding behavior.

This paper presents crease patterns unfolded by the inflation of a vein. Out of the characteristics discovered, only the snapping behavior can trigger the folding mechanism. While this paper is not focusing on the technical folding mechanism, other research groups are currently working on it. For example, an interdisciplinary research team in Germany, from the Institute for Building Structures and Structural Design, University of Stuttgart (ITKE), the Institute for Textile and Fiber Technologies, University of Stuttgart (ITFT), the German Institutes for Textile and Fiber Research Denkendorf (DITF), and the Evolutionary Biology of Invertebrates, Institute of Evolution and Ecology, University of Tübingen (EVE), has developed a product called Flexafold for an adaptive facade shading system [16]. Multiple veins can be individually actuated to partially fold and fully unfold the pattern. 

Because of the great diversity of insect species with foldable wings, current biological research is constantly discovering new folding and unfolding mechanisms along with unknown crease patterns. While some mechanisms are present throughout an entire order, others are specific to certain species. Therefore, the study and classification of trends in insect wings is not trivial and the scope of the discoveries is always subject to be modified. Throughout the known collection of insect wings, different mechanisms have been identified to fold the wings. For example, Coccinellidae of the order Coleoptera move their body back and forth to push their wings under the elytra [14]. Other Coleoptera use muscles located in their thorax to close the angle at the junction of the insect’s body and wings [17]. In Dermaptera, the wing folding is believed to be a result of stored energy in the patches of resilin [18].

Instead of bringing forward a specific pattern for a specific application, this paper displays parameters used to analyze a variety of folding patterns with the objective of showcasing their properties. With an application in mind, one pattern’s property can be seen as an advantage or a disadvantage. This approach is designed to increase the range of application, since different applications will have different constraints and parameters to optimize. For example, the design of a satellite will focus on the ratio between the folded and unfolded surface areas to make it as packable as possible during transportation. Parameters focusing on the snapping or folding mechanisms may not be as important because most deployable objects in space only unfold once. Unfolding large parts of a satellite, such as solar panels, with only one vein would greatly reduce the energy used to open it. The satellite will just need a system to push a liquid or gas through the vein. This vein does not have to stay inflated because of the low air friction in space. The authors have the assumption that this inflation could happen somewhat easily when the satellite is detaching itself from parts only used for transportation. For example, the fuel or air excess could be pushed in the vein before excreting it. In another environment, the goal may be to maintain the unfolded state against external forces. This could be done by keeping the vein inflated or by using a hardening liquid in the vein that could be disposed of over time, similar to the meconium in Lepidoptera [12]. To further reduce energy consumption, passive actuation through the use of shape memory alloys has also been studied as a one-time unfolding mechanism [19].

Another avenue for innovation inspired by insect wings is emerging from the potential of having non planar folded states. As seen in the Materials and Methods Section 2.1, the external surface of an insect’s body usually has a positive gaussian curvature, which means that the wings are not flat in the folded state. This curved folded state makes these patterns complicated to directly apply to humans’ applications, since a successful folded state is usually seen as flat. On the other hand, it opens up a large potential for applying foldable structures to non-flat environments.

In order to use the patterns presented above for a specific application, they will need to be adapted to be as efficient as possible for the targeted application. During this process, the designer may run into similar issues as the authors, such as facing vertices after a mirror transformation. In this regard, the apparent failures in our investigations were not discarded but presented in this paper. With a specific application in mind, materials and hinge mechanisms will also need to be taken into consideration.

The study of the geometry as an abstracted characteristic of folding patterns identified future areas of research: potential applications of the blocking behavior observed in some of the patterns, consequences of materials and hinge mechanisms on the folding structure, and the use of digital simulations to calculate internal and external forces in the folding material.

## 5. Conclusions

In conclusion, this paper studied the wing folding actuation mechanisms and crease patterns across numerous species and orders. The goal was to look at the implementation of veins as an actuator to unfold crease patterns both inspired by insects’ wings and adapted from the Miura-ori pattern. Instead of focusing on a specific application, this paper presents a range of folding patterns along with parameters to analyze them. With an application in mind, one could look at the properties of a folding pattern and depending on the parameters of importance, choose the most efficient pattern. These patterns should then be adapted to fully suit the desired application.

## Figures and Tables

**Table 1:** Results of the analysis performed on the folding patterns generated to study their underlying properties. If the pattern could not be fully folded, no data could be collected for the surface area difference, maximum number of overlapping sheets and exactly stacked edges as measurements from the folded state are needed.

	Properties	Number of Flat States	Number of Stable States	Number of Unit Cells	Surface Area of Folded/Unfolded States (%)	Maximum Number of Overlapping Sheets	Maximum Number of Exactly Stacked Edges	Other Properties
Patterns								
**Insect Wings Inspired**								
#101		2	1	5.25	9.8	21	4	/
#102		2	2	8.75	6.4	35	8	snapping
#103		1	1	15.75	/	/	/	/
#104		2	1	5.25	10.7	21	4	/
#105a		1	1	10.50	/	/	/	/
#105b		2	2	8.75	7.8	35	2	snapping
#106		1	1	15.75	/	/	/	/
#107		1	1	6.75	/	/	/	/
#108		1	1	11.25	/	/	/	/
**Miura-ori Variations**								
#201		2	1	8.75	4.3	35	15	/
#202		2	1	8.75	4.7	35	15	/
#203		2	1	8.75	6.8	35	15	/
#204		2	1	12.25	4.3	42	21	/
#205		2	1	12.25	6.0	28	14	/
#206		2	1	12.25	5.3	42	7	curvature
